# Chagas disease in the context of the 2030 agenda: global warming and vectors

**DOI:** 10.1590/0074-02760200479

**Published:** 2022-05-27

**Authors:** Rita de Cássia Moreira de Souza, David E Gorla, Marcia Chame, Nicolas Jaramillo, Carlota Monroy, Lileia Diotaiuti

**Affiliations:** 1 Fundação Oswaldo Cruz-Fiocruz, Instituto de Pesquisas René Rachou, Belo Horizonte, MG, Brasil; 2 Universidad Nacional de Córdoba, Instituto de Diversidad y Ecologia Animal, CONICET, Córdoba, Argentina; 3 Fundação Oswaldo Cruz-Fiocruz, Centro de Informação em Saúde Silvestre, Rio de Janeiro, RJ, Brasil; 4 Universidad de Antioquia, Instituto de Biología, Medellin, Colombia; 5 Universidad de San Carlos de Guatemala, Facultad de Ciencias Químicas y Farmacia, Guatemala City, Guatemala

**Keywords:** Chagas disease, triatomines, global warming, 2030 Agenda

## Abstract

The 2030 Agenda for Sustainable Development is a plan of action for people, planet and prosperity. Thousands of years and centuries of colonisation have passed the precarious housing conditions, food insecurity, lack of sanitation, the limitation of surveillance, health care programs and climate change. Chagas disease continues to be a public health problem. The control programs have been successful in many countries in reducing transmission by *T. cruzi*; but the results have been variable. WHO makes recommendations for prevention and control with the aim of eliminating Chagas disease as a public health problem. Climate change, deforestation, migration, urbanisation, sylvatic vectors and oral transmission require integrating the economic, social, and environmental dimensions of sustainable development, as well as the links within and between objectives and sectors. While the environment scenarios change around the world, native vector species pose a significant public health threat. The man-made atmosphere change is related to the increase of triatomines’ dispersal range, or an increase of the mobility of the vectors from their sylvatic environment to man-made constructions, or humans getting into sylvatic scenarios, leading to an increase of Chagas disease infection. Innovations with the communities and collaborations among municipalities, International cooperation agencies, local governmental agencies, academic partners, developmental agencies, or environmental institutions may present promising solutions, but sustained partnerships, long-term commitment, and strong regional leadership are required. A new world has just opened up for the renewal of surveillance practices, but the lessons learned in the past should be the basis for solutions in the future.

In September 2015, the 2030 Agenda for Sustainable Development was concluded during the United Nations Sustainable Development Summit, an initiative that follows the agreements of the RIO+20 Conference, held in Brazil.^1^ The agenda is a global action plan based on five central components to the challenges of sustainability - protecting people, planet, guaranteeing prosperity and peace through partnership.

From the 2030 Agenda, the Sustainable Development Goals (SDGs) were established. The period for its implementation runs from 2016 to 2030. The SDGs established 17 goals and 169 targets, with qualitative, quantitative and financial indicators.^1^

In this perspective, control of Chagas disease, marked by complex environmental, social and cultural determinants,^2^ transcends the biological relationships *Trypanosoma cruzi*-host, going through different objectives of the 2030 Agenda. It is not possible to think about the future of triatomine control without a brief tour on the natural history of Chagas disease.

The origin of Chagas disease can be proven by the presence of *T. cruzi* in prehistoric human material. Classical pathologies were found in mummified bodies and molecular techniques found the parasite’s DNA in Chilean and Peruvian mummies dated between 9,000 - 500 BP.^3^ ^,^ ^4^ ^,^ ^5^ ^,^ ^6^ In Brazil, *T. cruzi* DNA was demonstrated in a mummified body dated 540 ± 40 BP, in the Vale do Peruaçu in the State of Minas Gerais.^7^ The oldest meeting of Chagas disease reported outside of South America is the identification of *T. cruzi* DNA in a mummified man’s body dated 1,150 years old, found in the Texas-Coahuila region, USA, with megacolon, a characteristic pathology of the disease.^8^

Although thousands of centuries of colonisation have passed the precarious housing conditions, food insecurity, lack of sanitation, the limitation of surveillance and health care programs still puts 70 million people at risk, causing the death of 12,000 and 30,000 new infections per year worldwide.^9^ ^,^ ^10^ ^,^ ^11^ Chagas disease is still the disease of poverty in rural areas and for those who have no political voice and strength.^12^ ^,^ ^13^

Considering the commitments made by countries to reach SDGs by 2030, concrete advances and benefits for the control and prevention of Chagas disease can be expected. The analysis of SDGs, and the scientific data available allow us to identify that of the 17 SDGs, 14 contribute positively to the reduction of Chagas disease, two contribute indirectly and only for SDG 14 we are unable to identify an impact relationship on the disease (Table). Thus, we propose a reflection based on data on the occurrence and control of triatomines in the context of South and Central America, as elements for thinking about the near future.

**TABLE:** Impact of sustainable development goals (SDGs) in Chagas disease

SDGs can affect Chagas disease?		How	Why	References
SDG 1	End poverty in all its forms everywhere	↑	Chagas disease mostly affects poor and unassisted people. According WHO “Chagas disease is a proxy for poverty and disadvantage: it affects populations with low visibility and little political voice, causes stigma and discrimination, is relatively neglected by researchers, and has a considerable impact on morbidity and mortality”.	126 12 13 2 127
SDG 2	End hunger, achieve food security and improved nutrition and promote sustainable agriculture	↑	Oral transmission of *Trypanosoma cruzi* associated with food / beverage consumption is an important path of infection in South America that from 2000-2010 recorded 70% of the cases in the Amazon region. This type of transmission is basically related to improper handling by extractive communities and trade of regionals fruits such as açaí and bacaba. Other cases have also been reported for the consumption of sugar cane. The production of goats and sheep represents an expressive part of the economic activity of rural communities in South America, as well as the raising of pigs. Usually these animals are in corrals in the peridomicile and are food source of the triatomines, vectors of Chagas disease. The infestation of these insects is an impact factor on production.	128 129 130
SDG 3	Ensure healthy lives and promote well-being for all at all ages	↑	Chagas disease significantly affects the countries of South America where premature death leads to the loss of thousands of working days and a billion dollar impact on productivity. Health care costs reach more than half a billion a year and the migration of infected people to non-endemic areas, such as Europe, requires specific resources for this disease in these areas. Chagas’ disease is highly prevalent in rural areas. A study in Brazil from 2000 to 2010, with 54,236 patients, indicates greater involvement and mortality rate among men. Most patients were over 60 years old (Nóbrega et al.). The improvement of housing in some areas and lower fertility may be one of the causes of the aging of the disease, which translates into low infection among young people and an increase in the cause of death in the elderly in these areas.	131 132 133 134
SDG 4	Ensure inclusive and equitable quality education and promote lifelong learning opportunities for all	↑	Little or incorrect information is one of the fundamental aspects that compromise health promotion and actions for the prevention and control of zoonoses. In the case of Chagas’ disease, the knowledge of the vector species, their behavior, habitat and form of transmission of *T. cruzi* are essential for people to recognise the risk of infection and assist in entomological surveillance. Likewise, decision makers need to have the same knowledge to implement public policies and effective and efficient actions. The use of videos and films, iconographic atlases and vector albums and the collaboration of society are useful tools for everyone to become responsible in controlling the disease.	135 136 137
SDG 5	Achieve gender equality and empower all women and girls	↑	Chagas disease can be a congenital disease. The WHO points out that the elimination of congenital transmission depends on early diagnosis in infected pregnant women, newborns and immediate treatment. As the effectiveness of antiparasitic treatment is more frequent, faster and with fewer adverse reactions in girls under 19 years of age, it is important to promote the diagnosis before pregnancy, especially in non-endemic areas. The adoption of positive attitudes is one of the objectives of intervention programs for Chagas’ diseases, which must adopt different communication strategies between the sexes. Those that increase women’s knowledge about prevention and control of Chagas’ disease can contribute to increase the levels of adoption of good practices. For men, on the other hand, a strategy must involve practical and collaborative activities, differences that must be used in planning programs.	138 139 140
SDG 6	Ensure availability and sustainable management of water and sanitation for all	→	Good practices in food preparation, transportation, storage and consumptiom are recommended as a supporting actions to prevent the risk of oral *T. cruzi* infection.	141 142
SDG 7	Ensure access to affordable, reliable, sustainable and modern energy for all	→	Access to energy allows the maintenance of food and production of perishables, promotes comfort, quality of life and reduces manual labor, conditions that benefit extractive communities and rural producers in areas at risk of Chagas disease. Energy also promotes access to information through television and the internet that contribute to health education. On the other hand, home and street lighting can be an attraction factor for Chagas disease vectors and could increase the contact rates between vectors, humans and domestic animals.	143 144 145
SDG 8	Promote sustained, inclusive and sustainable economic growth, full and productive employment and decent work for all	↑	Food production, extractivism and changes in land use should include in their business plans the assessment of the impact of the activity on health and in Latin America in Chagas disease. They must consider the exposure and protection of the worker to the vector and the pathogen, environmental changes and biological communities that alter the natural cycle, the increase in the population density of humans and domestic animals attracted by economic growth, a scenario usually accompanied by precarious housing and conditions. Sanitary conditions, low capacity for surveillance and health care, factors that open alternative routes for infection by *T. cruzi*. The neglect in considering these factors led to outbreaks of Chagas disease due to oral infection.	146 147 148
SDG 9	Build resilient infrastructure, promote inclusive and sustainable industrialisation and foster innovation	↑	Efforts around the world to ensure food quality can be seen in advancing health standards and regulatory frameworks in several countries to prevent foodborne diseases. These advances include the control of oral transmission and expansion of Chagas disease. However, there is an urgent need for the sanitary surveillance system to be broad and efficient to identify potential risks, maintain risk analyses and control outbreaks of foodborne diseases worldwide.	149
SDG 10	Reduce inequality within and among countries	↑	Chagas disease is a social disease traditionally installed in poor communities with low assistance and political and economic inequalities. In the last 30 years it has expanded to the Amazon region with agricultural, urban, mining, livestock and timber extraction. The loss of territory by traditional and indigenous populations and the prospect of improving the quality of life and job offers in urban centers, inside and outside Latin American countries, stimulated the mobility of infected people coming from these areas, to other non-endemics. Migration to Europe and the United States (more than 300,000 legal immigrants) expanded the distribution of the disease. Although in many of these areas there is no possibility of maintaining transmission, the disease becomes a problem due to its morbidity and other forms of non-vector transmission.	150 2 151 118 152
SDG 11	Make cities and human settlements inclusive, safe, resilient and sustainable	↑	The maintenance of vector transmission of Chagas disease is closely related to human settements that provide habitat for several species of insect vectors. Adobe wall homes and corrugated metal roofing shelter triatomines vectors and subsistence living without drinking water sources contribute to the occurrence of Chagas disease. Although this scenario is still common in rural areas, colonisation and adaptation of vectors to urban constructions is increasing.	153 142 154
SDG 12	Ensure sustainable consumption and production patterns	↑	The consumption of açaí and other fruits and plants can lead to the risk of oral infection by *T. cruzi* if adequate sanitary measures are not observed and implemented in the production chain. This risk is low in traditional management in native areas and high in inadequate management in monocultures that expand the scale of production and degrade the forest structure.	155
SDG 13	Take urgent action to combat climate change and its impacts	↑	Triatominae, vectors of Chagas disease, occur in tropical and subtropical regions. For the establishment and population stability, these vectors need protection from climatic extremes, environments whose relative humidity does not exceed the annual average of 60% and a regular source of food (blood). Global climate and environmental changes can alter these parameters, increase temperature and expand the geographic distribution of these insects and make new habitats available.	156 118 116 157 117 106
SDG 14	Conserve and sustainably use the oceans, seas and marine resources for sustainable development	×	no impact identified	^ ^
SDG 15	Protect, restore and promote sustainable use of terrestrial ecosystems, sustainably manage forests, combat desertification, and halt and reverse land degradation and halt biodiversity loss	↑	Studies point out the impact of biodiversity loss on Chagas disease transmission and relate the lower richness of small mammals and the high infection in domestic dogs as a risk factor to Chagas disease transmission. This scenario seems to be consolidated after the human invasion of forest systems because the presence of domestic animals and synanthropic mammals, competent hosts of *T. cruzi*, promotes an increase in the abundance of vectors. In intact environments, the high diversity of species balances the distribution of the protozoan between competent and incompetent hosts (dillution effect).	121 120 119
SDG 16	Promote peaceful and inclusive societies for sustainable development, provide access to justice for all and build effective, accountable and inclusive institutions at all levels	↑	In South America and the Caribbean, many populations live in areas of armed conflict or social instability that hinder or even prevent vector control, the diagnosis and treatment of Chagas disease, among others. In these locations, the persistence of violence instills fear and abandonment of public power, leading thousands to cities and countries in search of security and quality of life.	158 159 160
SDG 17	Strengthen the means of implementation and revitalise the global partnership for sustainable development		Chagas disease is a tropical and subtropical disease that affects millions of people in several countries, especially poor and migrant people. Improving research and knowledge, sustainable economy, social justice, public policies, biodiversity conservation and surveillance and assistance and health actions can only be achieved with intersectoral partners and joint efforts.	161 162

↑ : advances in the objective positively impact the reduction of Chagas disease; → : advances in the objective indirectly contribute to the reduction of Chagas disease; × : no impact identified

**Brazil**

Brazil has played a major role in the development of methodologies for control of triatomines. The effective use of insecticides for elimination domestic populations of triatomines was first reported in the state of Minas Gerais.^14^ Until 1962, vector control had been a low priority in Brazil as in the other Latin American countries. The control program was managed at the national level,^15^ implemented in the whole endemic area of Brazil in the late 1970s and early 1980s, and maintained until 1999.

The control used BHC until 1986, when this insecticide was replaced with pyrethroids. The results were exceptional, with excellent impact on domestic triatomines populations.^16^ However, as a result of the transient action of the insecticide and the complexity of annexes, the response was not equally positive for control peridomestic populations of native species. These colonies are very close to the houses, representing a great risk: transmission may continue in close proximity to dwellers,^17^ and triatomines may displace between the different environments.^18^

Despite the authoritarian character of the control program at this time, it corresponds to the greatest advances in the implementation of entomological surveillance with the participation of communities in Brazil. Dias and Garcia^19^ experience in Bambuí, with educational initiatives conceived within Paulo Freire^20^ and Hortencia de Hollanda´s praxis,^21^ inspired an adaptation of this proposal to the surveillance of areas in the state of Minas Gerais.^22^ In 1990 Christopher John Schofield and João Carlos Pinto Dias reflected on the Brazilian success of eliminating *T. infestans* from large regions of Brazil, and dreamed that it would be possible in other areas of occurrence of this species. In Brasília, June 1991, the proposal was officially considered priority by South American countries.^23^ “The dream was over, because it is a reality now!” - Dias and Schofield commemorated.

The advances of the Southern Cone Initiative for the elimination of domestic populations of *T. infestans* were evident in all countries,^24^ but important foci still remained, especially in the Chaco region in Argentina, Bolivia and Paraguay.^24^ For Brazil, participation in such initiative consolidated the elimination of *T. infestans* from its residual foci, officially recognised by PAHO.^25^ The precipitated transference of the control activities from the federal to municipal level of health actions,^26^ nevertheless, resulted in discontinuity of the work in wide areas, especially because of the misconception that Chagas disease and vector transmission no longer existed in Brazil after the elimination of *T. infestans*.

Currently, in Brazil 66 native species of triatomines have been recognised.^27^ ^,^ ^28^ This challenging biodiversity includes species with evident capacity for colonisation the households and peridomestic annexes. In this scenario, the greatest challenge is to maintain surveillance in areas traditionally recognised as endemic, and to monitor other forms of transmission whose occurrence is increasing, especially the oral transmition, as well as species with high potential to invade the houses without colonisation.^29^

The classic endemic region in Brazil corresponds to the open environments of the Cerrado and the Caatinga.^16^ Native species of the Cerrado are of great importance in human transmission of *T. cruzi*. The domiciliation capacity of *Panstrongylus megistus* is clearly associated with deforestation,^30^ as occurred in Lassance at the beginning of the 20th century. It can sustain high prevalence rates of Chagas disease even in areas where *T. infestans* has never occurred.^31^

*Triatoma tibiamaculata* and *Triatoma vitticeps*, predominantly wild vectors,^32^ often invade the houses, owing to the active mobility of adult specimens, which eventually form small domestic colonies. Rates of infection of these vectors are frequently over 60%, in close association with marsupials and rodents.^33^ ^,^ ^34^ ^,^ ^35^ In fact, these triatomines are related to the endemicity of Chagas disease, albeit sporadically, as in the outbreak of oral infection in Santa Catarina, linked to *T. tibiamaculata*,^36^ and with the child’s death owing to oral transmission by infected *T. vitticeps*.^37^

*T. sordida* in the Cerrado,^38^ ^,^ ^39^ *T. pseudomaculata* and *T. brasiliensis* in the Caatinga,^40^ leave the wild environment and colonise the artificial environment, promoting persistent infestations, and can fully restore the colonies about a year after spraying.^41^ ^,^ ^42^ ^,^ ^43^

In the Amazon, the number of cases of Chagas disease has been increasing, mainly owing to the ingestion of food contaminated by *T. cruzi*.^29^ However, the possibility of classical vector transmission, through household invasion by highly infected triatomines, has also been reported.^44^ Brito et al.^45^ found that regional effects (Amazon/Cerrado), landscape (preservation/disturbance) and climate (temperature and rainfall), influence household invasion, at different levels, by *Rhodnius pictipes*, *R. robustos*, *R. neglectus* and *P. geniculatus*. Still, the invasion of houses by wild triatomines is a very common phenomenon.

In addition, especially in Argentina and Bolivia, there has been resistance of *T. infestans* to pyrethroid insecticides that have been widely used to control these vectors. They were initially praised for being biodegradable, virtually odorless, and used in much lower doses than those recommended for products in other categories (reduction of grams to milligrams/m^2^). Subsequently, *T. infestans* was found to be resistant to Fenitrothion and Fipronil,^46^ even in areas where these products had never been used. This finding demonstrates the wild nature of these profiles, regardless of the selection process resulting from pressure to use the products.

In Brazil, the susceptibility profile of *T. sordida* indicates the need for constant evaluations.^47^ In Ceará, all samples of *T. brasiliensis* under analysis were susceptible; the same result was found for *T. sordida* and *R. neglectus* in São Paulo.^48^ In fact, resistance is a complex and certainly multifactorial phenomenon,^47^ involving environmental aspects that must be considered for an analysis of the effectiveness of insecticide treatments. This assessment must be performed with a view to finding an adequate alternative, e.g., replacement of the insecticide with a product of a different class, to ensure that insects are indeed susceptible. At the same time, it is crucial that the application of the treatment should be evaluated for technical quality, as the persistence of colonies may be due to operational failure. This task requires a highly qualified team, and an agile notification system to indicate appropriate measures.

**Northern Andes**

*R. prolixus* is the main vector of *T. cruzi* in Colombia and Venezuela.^49^ There are records in Ecuador, but Abad-Franch et al.^50^ consider them doubtful. In the plains of the Orinoquía, large expanses of savannah shared by Colombia and Venezuela, sylvatic and domestic populations overlap. This region has the highest prevalence of Chagas disease in Colombia.^51^ In that region, *R. prolixus* colonises mainly the *Attalea butyracea* palm,^51^ ^,^ ^52^ probably due to the morphology of its crown, which is a very good environment for vertebrate’s nests. *R. prolixus* spread to agro-industrial crops of oil palm, *Elaeis guineensis*, which provides a similar habitat to *A. butyracea*.^53^ *E. guineensis* crops displaced the natural forest by hundreds of hectares and became the only refuge for *R. prolixus* and *R. robustus* which is morphologically indistinguishable from *R. prolixus*.^54^ This defies control planning since *R. robustus* does not colonises human domiciles, while *R. prolixus* does reinfest them after insecticide application.^55^ ^,^ ^56^ *R. prolixus* flies attracted by artificial light, up to 13 meters from its take-off base.^57^ Rendón et al.^52^ did not find eggs or nymphs inside the houses; but they found an infestation of 88.5% of *A. butyracea* palms with *R. prolixus*. This could indicate a stable zoonotic cycle where the ecosystem provides good conditions for insect development. However, the finding of children infected with *T. cruzi* despite the control of domicile colonies seem to demonstrate the importance of sylvatic vectors.^55^

*T. dimidiata* is of primary importance in Colombia and Ecuador. It is the cause of the urban transmission in the cities of Guayaquil and Portoviejo.^58^ Its importance in Venezuela is less clear, where it is considered a species of sylvatic habits.^59^ In the 1950s it was registered in northern Perú, but 50 years later the searches were negative.^60^ In Ecuador it has been found only in human homes; it is an altochthonous population that arrived by passive human transport from Central America.^50^ In Colombia, domestic and wild populations are found in the central-eastern Andean region of the country, circulating among houses, caves and rock accumulations; while in the west of the country, the populations are sylvatic associated mainly with *Attalea butyracea* palm.^61^ The populations of Ecuador and Perú are not geographically connected with those of Colombia and evolved as separate phylogenetic lineages.^62^

*R. ecuadoriensis* is along with *T. dimidiata* the main vector in Ecuador and northern Perú. It colonises dwellings and palm trees *Phytelephas aequatorialis* in central Ecuador, west of the Andes. At the southern border of Ecuador and northern Perú there is no *P. aequatorialis*, due to deforestation;^50^ but, *R. ecuadoriensis* is found in sylvatic squirrel nests (*Simosciurus nebouxii*) with an infestation rate of 13.6%.^63^

*T. maculata* is a species of secondary importance in Colombia and Venezuela. It colonises preferably peridomiciles, but frequently invades dwellings.^64^ Hernández et al.^65^ and Cantillo-Barraza et al.^66^ detected frequently the discrete typing unit (DTU) TcIb, very common in peridomiciliary vectors; but also, TcIDom infecting insects fed with human blood. This suggests, *T. maculata* could be in a transition from sylvatic to domestic environment and for this reason it should be subject to special surveillance.^64^

*Panstrongylus geniculatus* is a species of preferentially sylvatic habits; but it is frequently found in dwellings in all the countries of the Andean region.^50^ ^,^ ^67^ ^,^ ^68^ ^,^ ^69^ In Caracas, the Venezuelan capital, infected insects are often caught in highly urbanised areas and with evidence of human blood feeding. It has also been implicated in several outbreaks of oral transmission.^70^ As with *T. maculata*, *P. geniculatus* is believed to be in a transition from the sylvatic to the domestic environment and should be the target of special surveillance.^64^

The efforts of the “Initiative of the Andean Countries to Control Vectorial and Transfusion Transmission of Chagas disease” have been asynchronous and the results heterogeneous; but overall, it was possible to significantly reduce and, in many locations, eliminate transmission of the parasite.

In Ecuador, *T. dimidiata* infestation in the country’s coastal region was significantly reduced and the 100% screening of blood banks was achieved. In 2019, Ecuador set as a national objective the elimination of mother-to-child transmission of *T. cruzi*.^71^ But high rates of re-invasion of sylvatic insects and reinfestation by insecticide-resistant insects showed that vector control was not as effective an approach.^72^ In Ecuador, there is evidence of underreporting of cases, there is no systematic coverage of vector surveillance and control in a large area of the country, there is no program to control insect intrusion into the dwellings, and access to diagnosis is limited. Based in these facts Dumonteil et al.^73^ assert that Chagas disease has not been controlled in Ecuador.

In Colombia there are 66 municipalities certified free from the transmission of *T. cruzi*, 100% of donated blood is screened, there are protocols to attend and treat the chagasic patient, there is a plan for vector surveillance, there is a home improvement plan, and case fatalities down 48%.^74^ ^,^ ^75^ But Chagas disease continues to be a public health problem, with the highest prevalence in the Orinoquía region (7.0%)^51^ where domestic and sylvatic cycles overlap.^52^ ^,^ ^55^

Peru focused on eliminating *T. infestans* from the south of the country; but there is no systematic control activity in the north, directed against *R. ecuadoriensis*.^60^ *R. ecuadoriensis* infests dwellings, but also the white-naped squirrel nests, *Simosciurus nebouxii*, in northern Peru and southern Ecuador, an area of dry forest with similar ecology and land use, to consider when planning Chagas disease control.^76^

Venezuela demonstrated from 1960s to 1990s that it is possible to eliminate Chagas disease as a public health problem. In thirty years, the endemic area was reduced by 50%, the prevalence in blood banks remained below 1%, the seroprevalence rates decreased from 25.5% to 8.1% and the rates of infestation by *R. prolixus* fell from 80-60% to 4.0-1.6%.^77^ But for 2009, there was a re-emergence of the disease which main causes were the decentralisation of the health system, the reinvasion of homes by sylvatic *R. prolixus*, the increasingly frequent presence of *P. geniculatus* in homes, the involvement of *T. maculata* in oral transmission.^77^ ^,^ ^78^ Between 2007 and 2018, 16 outbreaks of oral Chagas disease were recorded across the country.^80^ Currently, Chagas disease is not controlled in Venezuela because there is a large variety of zoonotic cycles, it is increasingly urban and there are no solid public health policies.^79^ ^,^ ^80^

The effects of climate change in the northern Andean region on vector-borne anthropozoonoses are related to deforestation and accelerated and disorderly urbanisation due to migrations from the countryside to cities. Deforestation and urbanisation are strongly associated with the reduction of genetic variability of vectors and hosts, which increases the frequency of contacts between vectors and humans.^81^ ^,^ ^82^ The effects of climate change may not increase the risk of *T. cruzi* transmission by *R. prolixus* in a region; however, an increase in temperature in areas above 1140 masl will extend the vector’s habitability range in large areas in the next 13 years.^83^

**Central America and México**

With the elimination of the introduced vector, *R. prolixus*, from the Mesoamerican region, the opportunity was open for other native triatomine species to become the main vectors of Chagas disease. These species show high movement capacity and also seasonal annual movements that facilitate the re-infestation.^84^ ^,^ ^85^

The description of the triatomine Central American species was recently reviewed.^86^ In Mexico, the divergence of triatomines involves more than 40 species, with the *Phyllosoma* complex (*T. pallidipennis*, *T. longipennis*), *T. dimidiata*, and *T. barberi*, being abundant. At least 19 species have been found in human dwellings and infected with *T. cruzi*. Mexican triatomine vectors have urbanised in most regions of the country (88%), demonstrating a high tolerance to human-modified habitats.^87^ Due to the great diversity observed in triatomines, studies focused on understanding their biology, distribution, habitats, and relationship with human dwellings, which is necessary to establish vector control measures for each of the native species.

*T. dimidiata* and *T. barberi* are the main domiciliated vectors.^88^ The *Phyllosoma* complex includes morphotypes with chromatic and genetic varieties, with capacity of natural breeding with fertile descendants, which means individuals belonging to the same species; however, each variation has its own specific adaptation to environments and transmission capacity.^89^ The complex includes several species (*T. bassolsae*, *T. longipennis*, *T. mazzottii*, *T. mexicana*, *T. pallidipennis*, *T. picturata*, and *T. phyllosoma*), some are associated to peri-domiciliary environments of stone fence human constructions.^90^

*Rhodnius pallescens* is the main vector in Panama, but it is also found in limited areas in Costa Rica and Nicaragua (OPS/DPC/CD/273/03). Although it is a sylvatic vector related to palm trees (*Attalea*, *Acromia*), it is a visitor of houses near the palm trees, feeding on humans, marsupial and squirrels. Since it is a periodical visitor of the houses, the traditional insecticide application has limited efficacy, and the need for new integrated control measures is clear. The destruction of native palm trees is related to an increase of vector abundance.^91^ Introduction of massive oil palms plantations (*Elaeis*) showed that human businesses are building ideal nests for *Rhodnius* reproduction.^92^ Generalist mammals (*Mus musculus*, *Sus scrofa*) and marsupials favors the *T. cruzi* infection in those oil palm plantations.^93^ Control of *Rhodnius* species coming from palm trees requires a specific and new innovative control design and awareness of the management of the palms.

*T. nitida* is one of the triatomines with the longest life span, up to 897 days.^94^ It has been reported as capable of colonising houses with domestic colonies and lives in highlands with differences in morphological features.^95^ It is also found in sylvatic forest with pines and oaks, which are used as firewood, bringing the bugs to the house surroundings. It is easy to control and has low transmission capacity due to its defecation pattern. *T. ryckmani* is mainly a sylvatic vector living in dry forests in cactus and bromelias.^96^ For 10 years, it has been found visiting houses and colonising chickencoops. Several droughts and the destruction of dry forests are pushing the vector to move to manmade facilities; however, the species has not been found naturally infected *with T. cruzi*.^97^

*T. barberi* is found in the highlands of México as one of the main domiciliated vectors. Defecation is fast after the blood meal and the species is mainly collected in wall cracks near beds, there are no studies of the sylvatic environment for the species.^90^

The more deforested areas in Guatemala are the ones with more triatomines infestation and long-term control requirements. An alternative now being implemented is specific forest restoration with endemic regional plants. It is the case in Jutiapa, Guatemala, where it started with inventories of native plants species and specific selected seed nurseries. Actions for the vector control seldom include forest restoration, thus it is the first attempt to correlate Chagas disease and forest as an integral developmental program.^86^

Since we are expecting a future scenario of constant and rapid changes, strong efforts to sustain and improve surveillance procedures are crucial, especially when change in environmental factors or land use may favor the presence of one of the sylvatic triatomines into human dwellings. The most effective tool is the community surveillance, but to preserve the community interest in sending bugs to the health system, the answer to the communities has to be constant and permanent from the health personnel.

**Climate change and Chagas disease**

As cold blooded organisms, insect physiological and population responses depend strongly on temperature for reproduction, survival and dispersal. An environmental temperature increase would potentially lead to an expansion of the geographic distribution of vectors to areas that at present are too cold for their requirements. A temperature increase would increase the metabolic rate of parasites, shortening the infective periods, leading to the installation of a pathogen transmission cycle in warmer areas. With a simplistic ellaboration, a global increase in temperature would mean a world with expanded distribution of insect vector species and disease transmission into temperate areas. However, there has been considerable debate as to whether a global risk from vector-borne diseases will be impacted by climate change,^98^ ^,^ ^99^ although the discussions mostly ignored the biological diversity of vectors and vector-borne diseases.^100^

Simplistic predictions on climate change “diverts attention from the true reasons for the recrudescence of vector-borne diseases, including large-scale resettlement of people (often associated with major ecological change), rampant urbanisation without adequate infrastructure, high mobility through air travel, resistance to antimalarial drugs, insecticide resistance, and the deterioration of vector-control operations and other public-health practices”.^101^ Although climate is changing, other environmental and anthropogenic drivers can influence the spread of vector borne diseases. Further studies are necessary to better understand the phenomenon, and implement adaptive strategies to protect human health.^102^

There is increasing evidence of recent climate change impacts on some vector-borne diseases but for the most part, observed data series are too short, and impact of climate - independent factors too great, to confidently attribute a generalised changing risk to climate change. Changes in climate include increasing temperatures and variability, changes in the rainfall patterns and increasing frequency and severity of extreme weather events. On vector-borne diseases the expected results are changes in geographic seasonal patterns and risk levels of disease occurrence, changes in the dynamics of host communities, dispersal into new suitable environments, and impact of a wide range of human behavioural and socioeconomic factors on risk of exposure to infection.^103^

During the last years, the increasingly easy access to and availability of historical and future-projected climatic data, powerful tools for data analysis and powerful hardware to process large amounts of data, encouraged the explosive production of papers on the effects of climate change on the distribution of vector species. For the case of Chagas disease vectors, projections towards 2050 and 2070 expect changes in the geographic distribution of triatomines.^104^ ^,^ ^105^ ^,^ ^106^ Important as they are, temporal projections have to wait some time to be verified. Health systems needs are planned for the short or medium term. For the case of Chagas disease, this would mean continuous monitoring of the presence of vector species in the vicinity of human houses, either on urban, periurban or sylvatic environments, as there is now enough accumulated evidences that under any of those environments the transmission of *T. cruzi* is possible.

For the case of the most important non-native vectors of Chagas disease, the effects of climate on disease transmission may be obscured, because vectors are relatively buffered against weather and climate as they live almost exclusively inside houses. The potential change in the *T. infestans* population increase rate and on *T. cruzi* transmission risk based on the feeding frequency of *T. infestans* under scenarios of temperature increase in Argentina was analysed.^107^ The study focused on the effect of temperature increase over the population growth rate of *T. infestans* at low population abundance (when growth rate is highest) and on the *T. cruzi* transmission risk, associated with its dependence on the feeding frequency of the vector (dependent on temperature).^108^ The strong seasonality of temperature in different regions of Argentina allows a positive population growth rate of vector population during the coldest part of the year (July) only on a small part north of the country. The predictions showed that an average temperature increase between 2-4ºC would result in an important expansion of the region where the vector population will show a positive rate of population increase during winter.

More recently, ecological niche modeling studies showed that minimum temperature is one of the main climatic variable determining the distribution of *T. infestans*.^109^ ^,^ ^110^ Important as they are to understand the population ecology of the species, these studies are limited when predictions are only based on the climatic effect. A species like *T. infestans*, so closely associated with the human habitation, depends not only on temperature but on many other variables, including sociological, demographics and cultural ones.

Sometimes, a complex network of interactions (including climate change) produce a change in the epidemiological landscape of Chagas disease. The north of Córdoba province in central Argentina had very high house infestation, with domestic populations of adult *T. infestans* showing > 80% *T. cruzi* infection in 1986.^111^ After two decades, the area saw a 30% west-ward increase in rainfall, the iruption of the international market for soy bean, a technological innovation that allowed soy bean drought-varieties to be implanted in the area, the elimination of the natural vegetation cover, the expulsion of the original human population and the local disappearance of *T. infestans* populations.^112^ Locally, Chagas disease is not a problem anymore, simply because it was transported with the original human communities to the slum-villages at the outskirts of the big cities of Argentina.

The global process of rural-urban migration that in Latin America started in the 1940s reduced the number of rural communities living under housing conditions that were ideal for the development of *T. infestans* populations. Approximately 80% of Latin Americans will live in cities by 2025 (56.4% in 1970 and 79.5% in 2010).^113^ Within the area where *T. infestans* populations are still occurring, this migration process is active at present. Besides the reduction of the number of houses that could be colonised by the vector, this migration process lead to *T. infestans* population to colonise houses in cities, sometimes associated to pigeons, as in the city of San Juan (Argentina), or domestic animals (guinea pigs) as in Arequipa (Peru).

Field records collected 20 years apart (1986-2006) in northern Minas Gerais (Brasil), showed the colonisation of the area by *T. pseudomaculata* and a decrease of mammal fauna, associated with the increase of land surface temperatures because of deforestation.^114^ A similar situation was reported for the Jequitinhonha Valley, where deforestation was associated with an increase of *T. pseudomaculata* colonisation replacing *P. megistus* as dominant species. *T. pseudomaculata* is associated with drier environments than the ones where *P. megistus* is usually found.^115^

Vector control failures are sometimes justified by the climate change effect. Often, this argument has nothing to do with climate change, but it is a consequence of the amount of resources dedicated to the health system. For the case of triatomine vectors, we would not expect changes in the epidemiology of the disease as a direct consequence of climate change in the short term. We do expect important changes in the disease epidemiology as a consequence of the more or less strong environmental changes produced by deforestation, land cover changes and unplanned urbanisation. In this dynamic world, making predictions is not easy. However, we know that a strong and sustained vigilance system is one of the best strategies to navigate through unknown waters. This is not new, but vigilance and surveillance systems for triatomines have been weakening after the successful stories of the Latin American Initiatives. Nowadays, with better knowledge about triatomine vectors, better information and communication technologies, there is a chance to have control of the community health in a future uncertain world.

Considering the commitments made by countries to reach SDGs by 2030, concrete advances and benefits for the control and prevention of Chagas disease can be expected. The analysis of SDGs one by one, and the scientific data available allow us to identify that of the 17 SDGs, 14 contribute positively to the reduction of Chagas disease, two contribute indirectly and only for SDG 14 we are unable to identify an impact relationship on the disease (Table).

The SDGs that deal with the reduction of poverty and hunger (SDGs 1, 2), food security and production (SDGs 2, 12); health and equality (SDGs 3, 10), sanitation and housing (SDGs 6, 11) have an obvious impact on the driving forces that determine Chagas disease. However, ecological and epidemiological studies indicate that the other SDGs have a relevant impact on reducing parasitosis. Efforts and commitment to natural areas maintenance (SDG 15) and the fight against global climate change (SDG 13) play a fundamental role in reducing the expansion of the geographic distribution of triatomines.^116^ ^,^ ^117^ ^,^ ^118^ The conservation of forests and natural areas prevents the loss of biodiversity and the dispersion of vectors to peri-urban and urban areas.^119^ ^,^ ^120^ ^,^ ^121^ The reduction of these areas promotes impacts for the emergency and transmission of infectious diseases.^122^ Commitments to promote fair working conditions, production, consumption and sustainable infrastructure (SDG 7, 9, 12) will benefit traditional populations that use and exploit natural resources in their economic activities.^123^ Likewise, partnerships between countries (SDG 17), financial mechanisms and the transfer of clean technologies are expected to reduce not only environmental impacts, but generate new jobs and peace (SDG 16). Populations living in degraded areas, occupied by civil conflicts and social instability suffer from limited health care, education, violence against women, children and the elderly, in a continuous cycle of vulnerabilities.

While the 2030 Agenda and the SGDs bring hope for a more sustainable world, the pandemic scenario COVID-19 poses new challenges for health surveillance. Field actions for entomological control have been reduced as well as the maintenance of health care services in communities due to social isolation.^124^ In many countries, these actions have already been reduced due to the success of previous disease control programs.^10^ However, new possibilities should be encouraged, especially those that use digital technologies. Mobile phones, app and the internet allow people to contact public health agencies in their homes. Through the network they can inform the presence of vectors,^125^ ask questions about symptoms, be assisted by a health professional, or receive diagnoses from tests or medical prescriptions. A new world has just opened up for the renewal of surveillance practices. But the lessons learned in the past should be the basis for solutions in the future.
